# Metabolite Profiling Reveals the Effect of Dietary *Rubus coreanus* Vinegar on Ovariectomy-Induced Osteoporosis in a Rat Model

**DOI:** 10.3390/molecules21020149

**Published:** 2016-01-26

**Authors:** Mee Youn Lee, Hyang Yeon Kim, Digar Singh, Soo Hwan Yeo, Seong Yeol Baek, Yoo Kyoung Park, Choong Hwan Lee

**Affiliations:** 1 Department of Bioscience and Biotechnology, Kon-Kuk University, Seoul 143-701, Korea; kkamlice@hanmail.net (M.Y.L.); festivalkim@naver.com (H.Y.K.); singhdigar@gmail.com (D.S.); 2 Fermented Food Science Division, Department of Agro-food Resource, National Academy of Agricultural Sciences, Rural Development Administration, Jeollabuk-do 565-851, Korea; yeobio@rda.go.kr (S.H.Y.); dunkbis@korea.kr (S.Y.B.); 3 Department of Medical Nutrition, Graduate School of East-West Medical Science, Kyung Hee University, Gyeonggi-do 446-791, Korea; ypark@khu.ac.kr

**Keywords:** osteoporosis, *Rubus coreanus* vinegar, ovariectomy, mass spectrometry, multivariate analysis

## Abstract

The study was aimed at exploring the curative effects of *Rubus coreanus* (RC) vinegar against postmenopausal osteoporosis by using ovariectomized rats as a model. The investigations were performed in five groups: sham, ovariectomized (OVX) rats without treatment, low-dose RC vinegar (LRV)-treated OVX rats, high-dose RC vinegar (HRV)-treated OVX rats and alendronate (ALEN)-treated OVX rats. The efficacy of RC vinegar was evaluated using physical, biochemical, histological and metabolomic parameters. Compared to the OVX rats, the LRV and HRV groups showed positive effects on the aforementioned parameters, indicating estrogen regulation. Plasma metabolome analysis of the groups using gas chromatography-time of flight mass spectrometry (GC-TOF-MS) and ultra-performance liquid chromatography quadrupole-TOF-MS (UPLC-Q-TOF-MS) with multivariate analysis revealed 19 and 16 metabolites, respectively. Notably, the levels of butyric acid, phenylalanine, glucose, tryptophan and some lysophosphatidylcholines were marginally increased in RC vinegar-treated groups compared to OVX. However, the pattern of metabolite levels in RC vinegar-treated groups was found similar to ALEN, but differed significantly from that in sham group. The results highlight the prophylactic and curative potential of dietary vinegar against postmenopausal osteoporosis. RC vinegar could be an effective natural alternative for the prevention of postmenopausal osteoporosis.

## 1. Introduction

Osteoporosis is a progressive bone disease characterized by reduced bone mass and density, which increase susceptibility to fractures [1]. The disease causes microarchitectural deterioration and alters the expression of a variety of proteins in bones [2]. This condition is more common in the elderly, but can affect men and women of all ages [3]. In particular, women are about five-times more likely to develop osteoporosis than men, because they have smaller and thinner bones than men do to begin with and they lose bone mass more rapidly after menopause [4]. About 200 million women worldwide are affected by postmenopausal osteoporosis [5]. Most women go through menopause between the ages of 45 and 55 years, which gradually reduces the levels of estrogen and progesterone [6]. Estrogen deficiency is associated with osteoporosis, because a reduction in estrogen level is often associated with increased rate of bone resorption in osteoclasts [7]. Hormone therapy is generally used to prevent and treat osteoporosis in addition to conventional medicines; however, it has some serious side effects in convalescents with thrombosis, hypertension and atherosclerosis [8]. Furthermore, bisphosphonates are mostly prescribed for the management of postmenopausal osteoporosis; however, these drugs are associated with esophageal cancer and jaw osteonecrosis [9]. Therefore, there is an urgent need for a broader perspective towards the discovery and development of health foods containing natural products that can effectively counter estrogen deficiency [10]. Milk [11], soybean [12] and vinegar [13] have been reported as health foods for managing osteoporosis. Vinegar is a sour traditional fermented food that is used in pickles, sauces and beverages, as well as in various food-processing procedures [14]. The health benefits of drinking vinegar are well known; it prevents cardiovascular disease [15], has an anti-obesity effect [16] and strengthens bones [17]. Several substrate materials used in the production of vinegar are known for their obvious health benefits [18,19]. One of the raw material substrates for vinegar production, *Rubus coreanus* (RC; Korean black raspberry, called *bokbunja*) from the raspberry family, contains large amounts of anthocyanin, which has antioxidant effects [20]. Additionally, RC-containing products, such as juice, tea, wine and jam, have been reported to show antiviral [21], anticancer [22] and anti-obesity [23] effects. Recently, there has been a renewed interest in RC products because of their anti-osteoporotic and bone-protective effects, such as enhancement of osteoblast function, induction of osteoclast apoptosis [24] and inhibition of bone resorption [25]. Although several studies have reported anti-osteoporotic effects of different RC products [26,27], detailed insights into their respective metabolic expressions and correlative bioactivities are unavailable.

Metabolomics is the evaluation of the levels of metabolites in cells, tissues, organs or biological fluids, which can produce a comprehensive quantitative metabolomic profile indicating the details of the underlying pathways. The metabolomic profile supplies vital information that can help understand not only the effect of food or drug intake on a disease, but also help diagnose an altered physiological state [28]. Recently, metabolite profiling of plasma from rat, mouse and human models was performed using mass spectrometry (MS)-based high-throughput techniques [29,30]. Ultra-performance liquid chromatography-hybrid quadrupole-time-of-flight mass spectrometry (UPLC-Q-TOF-MS) and gas chromatography-time-of-flight mass spectrometry (GC-TOF-MS) have been successfully applied to analyze and explain multiparametric metabolic responses of living systems to pathophysiological and environmental perturbations [31]. Moreover, emerging metabolomic technology provides a powerful platform for searching novel biomarkers and biochemical pathways to enhance diagnostic prognostication and therapy [32].

Therefore, to investigate the metabolomic vista of RC vinegar consumption on postmenopausal osteoporosis, we evaluated the dynamic cues of metabolites in the plasma from ovariectomized rats receiving RC vinegar. The likely effects of RC vinegar intake on the “osteoclast-osteoblast” activity equilibrium in the rat model were evaluated for different metabolomes by using high-throughput MS acquisitions methods (UPLC-TOF-MS and GC-TOF-MS) with multivariate statistical analyses.

## 2. Results

### 2.1. Body and Uterus Weight

**Figure 1:**
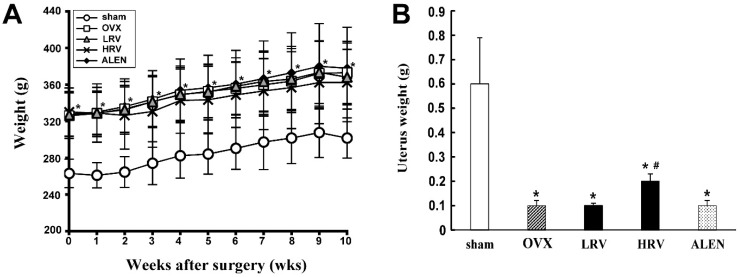
Effects of *Rubus coreanus* (RC) vinegar on body weight (**A**) and uterus weight (**B**) in ovariectomized rats. Data are expressed as the mean ± SD (*n* = 8). sham, sham-operated control; OVX, ovariectomized rat without treatment; LRV, ovariectomized rat treated with low-dose RC vinegar; HRV, ovariectomized rat treated with high-dose RC vinegar; ALEN, ovariectomized rat treated with alendronate. * *p* < 0.05 compared to the sham, # *p* < 0.05 compared to the OVX group.

Ten weeks after ovariectomy, the body weight of five groups was examined (Figure 1A). It tended to be higher in the ovariectomized rat groups (ovariectomized (OVX), low-dose RC vinegar (LRV), high-dose RC vinegar (HRV) and alendronate (ALEN)) than that in the sham group. Among the ovariectomized rat groups, there was no significant difference with respect to body weight.

Whereas the mean uterine weight in ovariectomized rat groups was significantly lower than that in the sham group (Figure 1B), among the treated groups, uterine weight in the HRV group was found to be significantly higher relative to the OVX and the rest of the groups.

### 2.2. Serum and Urinary Biochemistry

The serum and urine biochemical parameters are summarized in Table 1. Lipid profile analysis showed the AST and ALT activity of OVX rats to be slightly higher than that of the sham group after 10 weeks of treatment. Four biochemical markers (ALP, OC, DPD and NTx) for bone formation and bone resorption were also examined. Serum levels of ALP, a biomarker for bone turnover, were found to be significantly higher among the ovariectomized rat groups (OVX, LRV, HRV and ALEN) than that in the sham. The levels of OC, the major non-collagenous protein produced by bone osteoblasts, were slightly higher in OVX rats than those in other groups. Moreover, the serum OC levels were lower in sham and ALEN groups than those in rest of the groups.

Additionally, we evaluated the urinary biomarkers for bone resorption, DPD and NTx. These parameters were moderately high in LRV and HRV groups relative to OVX rats. In particular, the urinary DPD levels in the HRV group were comparable to those of the sham. In contrast, the levels of DPD and NTx were higher in OVX rats than those in the ALEN group. However, the serum levels for DPD and NTx were not found to be statistically significant among the different experimental and sham groups.

**Table 1:** Lipid profile parameter and biochemical markers of bone formation and resorption.

Parameters	Sham	OVX	LRV	HRV	ALEN
***Lipid profile***					
Serum AST (U/L)	127.5 ± 25.97	136.7 ± 26.09	134.6 ± 22.81	129.6 ± 27.97	112.5 ± 23.53
Serum ALT (U/L)	26.4 ± 5.24	29.1 ± 4.53	26.9 ± 8.68	25.9 ± 5.01	26.4 ± 4.63
***Bone formation***					
Serum ALP (U/L)	34.0 ± 6.80	45.7 ± 7.85 *	44.9 ± 5.22 *	42.6 ± 4.24 *	45.1 ± 7.66 *
Serum OC (ng/mL)	17.9 ± 3.77	21.6 ± 7.65	20.8 ± 6.47	20.0 ± 3.86	17.7 ± 5.46
***Bone resorption***					
Urinary DPD (nM BCE/mM Cr)	90.9 ± 47.04	70.2 ± 52.14	78.9 ± 53.90	92.8 ± 30.18	43.1 ± 13.35
Urinary NTx (nM BCE/mM Cr)	5.9 ± 2.19	5.9 ± 1.36	6.3 ± 1.06	7.5 ± 2.04	7.2 ± 2.57

Sham, sham-operated control; OVX, ovariectomized rats without treatment; LRV, ovariectomized rats treated with low-dose RC vinegar; HRV, ovariectomized rats treated with high-dose RC vinegar; ALEN, ovariectomized rats treated with alendronate. * There were statistical differences among the experimental groups by one-way ANOVA and Duncan’s multiple range tests (*p* < 0.05). Data are expressed as the mean ± SD (*n* = 8). AST, aspartate aminotransferase; ALT, alanine aminotransferase; ALP, alkaline phosphatase; OC, osteocalcin; DPD, deoxypyridinoline; BCE, bone collagen equivalent; Cr, creatinine.

**Figure 2:**
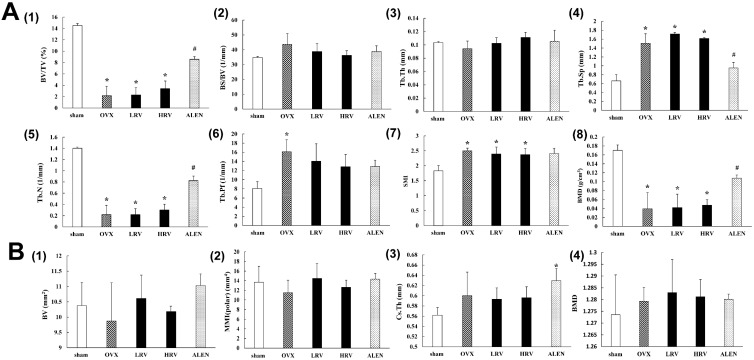
Monitoring of the trabecular bone (**A**) and cortical bone (**B**) parameters by the three-dimensional micro-CT analysis program. Trabecular bone parameters assessed (**A**): (A1) BV/TV, bone volume/tissue volume; (A2) BS/BV, bone surface/tissue volume; (A3) Tb.Th, trabecular thickness; (A4) Tb.Sp, trabecular separation; (A5) Tb.N, trabecular number; (A6) Tb.Pf, trabecular pattern factor; (A7) SMI, structure model index; (A8) BMD, bone mineral density. Cortical bone parameters assessed (**B**): (B1) BV, bone volume density; (B2) MMI, mean polar moment of inertia; (B3) Cs.Th, cross-section thickness; (B4) BMD, bone mineral density. sham, sham-operated control; OVX, ovariectomized rat without treatment; LRV, ovariectomized rat treated with low-dose RC vinegar; HRV, ovariectomized rat treated with high-dose RC vinegar; ALEN, ovariectomized rat treated with alendronate. * *p* < 0.05 compared to the sham, # *p* < 0.05 compared to the OVX group.

### 2.3. Micro-Computed Tomography Analysis

After 10 weeks of treatment, the trabecular bone and cortical bone parameters in the femur were analyzed by micro-CT (Figure 2). Significant differences were detected in the trabecular bone parameters (Figure 2A) between sham and ovariectomized rat groups. The levels of BV/TV (bone volume/tissue volume) (Figure 2A1), Tb.Th (trabecular thickness) (Figure 2A3), Tb.Sp (trabecular separation) (Figure 2A4), Tb.N (trabecular number) (Figure 2A5) and BMD (bone mineral density) (Figure 2A8) were marginally higher in LRV and HRV groups than those in OVX rats. In contrast, the parameters BS/BV (bone surface/tissue volume) (Figure 2A2), Tb.Pf (trabecular pattern factor) (Figure 2A6) and SMI (structure model index) (Figure 2A7) were moderately lower in LRV and HRV groups than those in OVX rats. These parameters showed dose-dependent recovery in LRV and HRV groups. Although the absolute levels of trabecular bone parameters in LRV and HRV groups were lower than those in the ALEN group, the pattern was similar to that observed with the positive control, ALEN. However, Tb.Sp (Figure 2A4) patterns for LRV and HRV groups showed the opposite tendency to those observed with ALEN.

The cortical bone parameters (Figure 2B), including BV, MMI, Cs.Th and BMD, were not significantly different. Additionally, three-dimensional micro-CT images of the trabecular bone and cortical bone are shown in Supplementary Figure S1. In the sham, the trabecular bone can be seen as a well-connected network, while in the OVX group, the trabecular bone withered and became separated. LRV, HRV and ALEN partly prevented the OVX-induced bone loss and improved the trabecular bone mass and microarchitecture after 10 weeks of treatment. No significant changes were observed in the cortical bone.

### 2.4. Analysis of Plasma Metabolite Profiles

**Figure 3:**
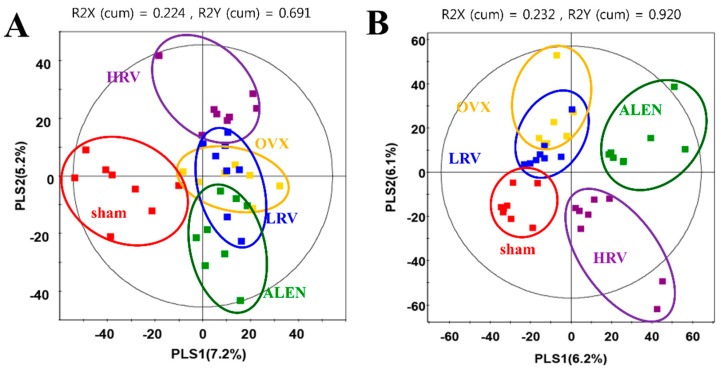
Partial least-squares discriminant analysis (PLS-DA) score plots derived from the GC-TOF-MS (**A**) and UPLC-Q-TOF-MS (**B**) data of ovariectomized rat groups. (■; sham-operated control, ■; ovariectomized rat without treatment, ■; ovariectomized rat treated with low-dose RC vinegar, ■; ovariectomized rat treated with high-dose RC vinegar, ■; ovariectomized rat treated with alendronate).

Rat plasma metabolite profiling was performed using GC-TOF-MS and UPLC-Q-TOF-MS. PCA (Supplementary Figure S2) and PLS-DA (Figure 3) analyses were used to distinguish the differences between the groups and to interpret the intrinsic similarities of each group from their chromatographic profiles. The PLS-DA score plots based on GC-TOF-MS (Figure 3A) and UPLC-Q-TOF (Figure 3B) showed clear differences between the five experimental groups. As shown in Figure 3A, metabolite profiles for sham and ovariectomized groups (OVX, LRV, HRV and ALEN) were separated by PLS1. The score plot of PLS-DA based on GC-TOF-MS data explained 12.4% of the total variability (PLS1: 7.2%; PLS2: 5.2%). Furthermore, PLS-DA models analyzed for UPLC-Q-TOF-MS data were separated for each group (Figure 3B). In the PLS-DA score plot, sham, LRV and OVX groups were distinguished from HRV and ALEN groups by PLS Component 1 (6.2%). In addition, sham, LRV and OVX were separated from HRV and ALEN by PLS Component 2 (6.1%). We selected significantly-different metabolites from the PLS-DA score plots of GC-TOF-MS and UPLC-Q-TOF-MS data, based on their variable importance in projection (VIP) value (>0.7) and *p*-value (<0.05). Consequently, 19 and 16 of the significantly-different metabolites were selected by GC-TOF-MS and UPLC-TOF-MS analysis, respectively.

### 2.5. Identification of Significantly-Discriminable Metabolites in Each Group

Significantly different metabolites from GC-TOF-MS and UPLC-Q-TOF-MS analyses were divided into five experimental groups based on the PLS1 and PLS2 components (Figure 3). The metabolites selected by (VIP) values (>0.7) in the GC-TOF-MS and UPLC-Q-TOF-MS analysis are presented in Table 2 and Table 3, respectively.

**Figure 4:**
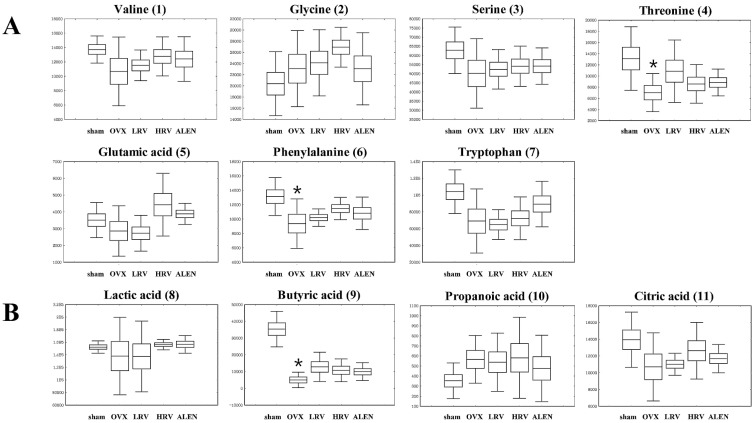

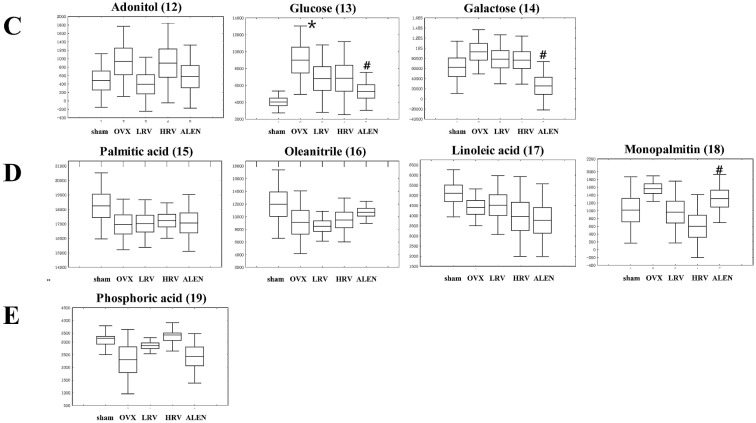
Box and whisker plot analyses of metabolites from GC-TOF-MS data among the five different rat groups. Amino acids (**A**); organic acids (**B**); sugars and sugar derivatives (**C**); fatty acids (**D**); and others (**E**). The numbers of the compounds in parentheses are the same as those referred to in Table 2. sham, sham-operated control; OVX, ovariectomized rat without treatment; LRV, ovariectomized rat treated with low-dose RC vinegar; HRV, ovariectomized rat treated with high-dose RC vinegar; ALEN, ovariectomized rat treated with alendronate. * *p* < 0.05 compared to the sham, # *p* < 0.05 compared to the OVX group.

In GC-TOF-MS analysis, a total of 19 compounds in five categories were identified, including seven amino acids, four organic acids, three sugars and sugar derivatives, four fatty acids and one “other” (Table 2). The 19 identified metabolites from five different experimental groups are presented by box and whisker plots, which were calculated from the peak area (Figure 4). Among them, butyric acid (**9**), phenylalanine (**6**), glucose (**13**) and tryptophan (**7**) were significantly different (*p* < 0.05) among the five experimental groups (shown as the *p*-value in Table 2). The level of the metabolites, except glucose (**13**), decreased in OVX rats and then gradually increased in LRV, HRV and ALEN groups (Figure 4).

**Figure 5:**
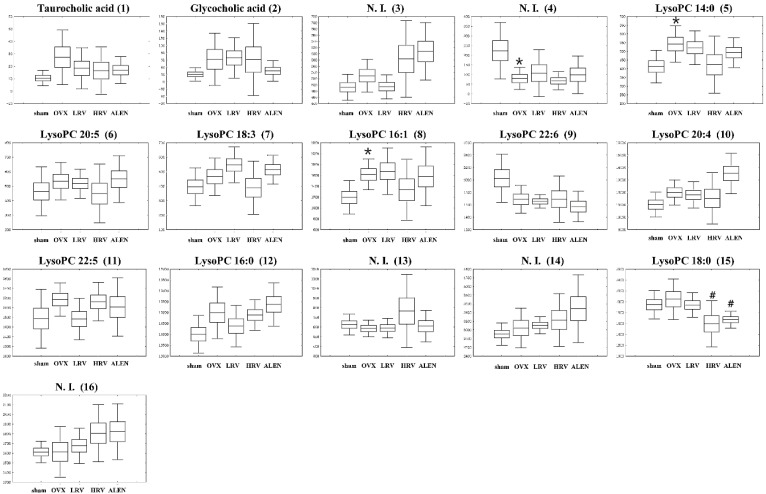
Box and whisker plots analyzed by UPLC-Q-TOF-MS of the five different rat groups. The results are the mean ± SD of triplicates from a representative experiment. sham, sham-operated control; OVX, ovariectomized rat without treatment; LRV, ovariectomized rat treated with low-dose RC vinegar; HRV, ovariectomized rat treated with high-dose RC vinegar; ALEN, ovariectomized rat treated with alendronate. * *p* < 0.05 compared to the sham, # *p* < 0.05 compared to the OVX group.

In the UPLC-Q-TOF-MS positive ion mode analysis, the five experimental groups were separated by PLS-DA, and their related metabolites are listed in Table 3. In total, 16 metabolites were identified as highly differential ones contributing to the five experimental group separations based on VIP values (>0.7). The discriminations between the different groups of rat plasma metabolites were putatively identified by matching their relative retention time, accurate mass, error (mDa) and molecular formula with those of the references and those listed in the Human Metabolome Database (HMDB). Sixteen metabolites, including two bile acids, nine lysophosphatidylcholines (lysoPCs) and five unidentified metabolites were selected. As shown in Figure 5, the relative contents of secondary metabolites among the five experimental groups are shown by box and whisker plots calculated using the peak area. Among them, six metabolites, lysoPC 16:0 (**12**), lysoPC 18:0 (**15**), lysoPC 20:4 (**10**), lysoPC 22:6 (**9**), and two unidentified metabolites (**3**), (**4**), determined to be associated with osteoporosis, were also identified in the rat plasma as the significantly-different metabolites (*p* < 0.05). Moreover, in the plasma phospholipids, concentrations of lysoPC 22:6 (**9**) were lower in OVX rats than those in the sham. Furthermore, LRV, HRV and ALEN groups exhibited slightly decreased levels of lysoPC 22:6 compared to the OVX rats. In contrast, lysoPC 16:0 (**12**), lysoPC 18:0 (**15**) and lysoPC 20:4 (**10**) slightly increased in the OVX rats. Plasma levels of lysoPC 16:0 (**12**) decreased in LRV, but increased in HRV and ALEN groups. In contrast, the other two lysophosphatidylcholines, lysoPC 18:0 (**15**) and lysoPC 20:4 (**10**), were detected moderately and increased negligibly, respectively, in the LRV group relative to the HRV group (Figure 5).

**Table 2:** Discriminant plasma metabolites identified using GC-TOF-MS in different rat groups.

No.	RT (min)	Tentative Metabolites ^a^	MS Fragment ^b^	*p*-Value	Derivatized	ID ^c^
***Amino acids***						
1	6.43	Valine	59, 100, 156, 218, 246	0.365	(TMS)_2_	STD
2	7.30	Glycine	86, 133, 174, 249, 276	0.280	(TMS)_3_	STD
3	7.79	Serine	100, 188, 204, 218, 278	0.385	(TMS)_2_	STD
4	8.04	Threonine	57, 86, 129, 219, 291	0.083	(TMS)_3_	STD
5	9.94	Glutamic acid	56, 84, 128, 246, 348	0.073	(TMS)_3_	STD
6	10.04	Phenylalanine	100, 192, 218, 266, 294	0.036	(TMS)_3_	STD
7	14.03	Tryptophan	59, 100, 202, 218, 291	0.039	(TMS)_3_	STD
***Organic acids***						
8	4.92	Lactic acid	45, 88, 117, 191, 219	0.724	(TMS)_2_	STD
9	5.55	Butyric acid	59, 115, 131, 205, 233	0.000	(TMS)_2_	MS
10	7.53	Propanoic acid	59, 133, 189, 205, 292	0.556	(TMS)_3_	MS
11	11.46	Citric acid	99, 133, 211, 273, 465	0.203	(TMS)_4_	STD
***Sugars and sugar derivatives***						
12	10.77	Adonitol	59, 103, 129, 205, 217	0.541	(TMS)_2_	STD
13	11.87	Glucose	59, 89, 103, 217, 307	0.081	(TMS)_4_	STD
14	12.03	Galactose	59, 103, 129, 205, 319	0.090	(TMS)_5_	STD
***Fatty acids***						
15	12.84	Palmitic acid	55, 95, 201, 269, 313	0.609	TMS	STD
16	13.13	Oleanitrile	55, 122, 136, 220, 263	0.397	TMS	MS
17	13.85	Linoleic acid	55, 129, 178, 262, 337	0.455	TMS	STD
18	15.90	Monopalmitin	57, 129, 203, 239, 371	0.121	(TMS)_2_	MS
***Other***						
19	11.60	Phosphoric acid	59, 211, 299, 357, 445	0.130	(TMS)_5_	STD

**^a^** The metabolites selected by the variable importance projection (VIP) value (>0.7); ^b^ MS fragmentation is the fragmentaion of tentative compound; ^c^ MS mass spectrum was consistent with those of NIST and in-house libraries; STD mass spectrum was consistent with that of the standard compounds; RT, Retention time; TMS, trimethylsilyl; ID, Identification.

**Table 3:** Discriminant plasma metabolites identified using UPLC-Q-TOF-MS in different rat groups.

No.	RT (min)	Tentative Metabolites ^a^	Measured MS (*m*/*z*)		HMDB Formula	Error ^b^ (mDa)	Adduct	*p*-Value
			Negative	Positive				
1	5.43	Taurocholic acid	514.2847	538.2816	C_26_H_45_NO_7_S	0.9	[M + Na]^+^	0.378
2	5.91	Glycocholic acid	464.3002	488.2986	C_26_H_43_NO_6_	−0.5	[M + Na]^+^	0.467
3	7.13	N. I. (1)	-	288.2910	-	-	-	0.015
4	7.63	N. I. (2)	-	357.2797	-	-	-	0.035
5	7.72	LysoPC 14:0	512.2922	468.3075	C_22_H_46_NO_7_P	0.0	[M + H]^+^	0.115
6	7.78	LysoPC 20:5	586.3132	542.3254	C_28_H_48_NO_7_P	0.7	[M + H]^+^	0.655
7	7.86	LysoPC 18:3	562.3149	518.3243	C_26_H_48_NO_7_P	−0.4	[M + H]^+^	0.099
8	7.98	LysoPC 16:1	538.3101	494.3202	C_24_H_48_NO_7_P	−0.3	[M + H]^+^	0.174
9	8.09	LysoPC 22:6	612.3307	568.3358	C_30_H_50_NO_7_P	−1.7	[M + H]^+^	0.035
10	8.27	LysoPC 20:4	588.3356	544.3422	C_28_H_50_NO_7_P	−1.0	[M + H]^+^	0.018
11	8.47	LysoPC 22:5	614.3473	570.355	C_30_H_52_NO_7_P	−1.0	[M + H]^+^	0.349
12	8.75	LysoPC 16:0	540.3259	496.3415	C_24_H_50_NO_7_P	0.1	[M + H]^+^	0.049
13	9.22	N. I. (3)	-	508.3765	-	-	-	0.383
14	9.33	N. I. (4)	-	228.2317	-	-	-	0.234
15	9.85	LysoPC 18:0	568.3609	524.3718	C_26_H_54_NO_7_P	−0.3	[M + H]^+^	0.580
16	10.44	N. I. (5)	-	282.2787	-	-	-	0.246

**^a^** Identified metabolites based on variable importance projection (VIP) value (>0.7); ^b^ Differences between observed mass and calculated mass. mDa stands for error in millidalton; RT, Retention time; N. I., Not identified; LysoPC, lysophosphatidylcholine. HMDB, Human Metabolome Database.

## 3. Discussion

The ovariectomized rats are commonly used preclinical animal models for postmenopausal bone loss in women [33]. In this study, we investigated the effects of RC vinegar on ovariectomy-induced osteoporosis in rats. After 10 weeks of ovariectomy, the mean body weight of the ovariectomized rat groups was significantly higher than that of the sham (Figure 1A). The weight gain in the ovariectomized rats is a commonly-observed phenomenon [34]. Menopause is associated with a propensity to gain weight, and estrogenic levels may influence body fat distribution [35]. This weight gain is relevant to the activation of estrogen receptors (ER). Estradiol, an estrogen sex hormone, functions through the intracellular receptors, ERα and ERβ [36]. Furthermore, estradiol increases insulin sensitivity, and therefore, estrogen replacement therapy in postmenopausal women has been shown to improve glucose homeostasis [34]. Wegorzewska *et al.* [37] observed that ER-knockout mice exhibit enhanced weight gain, compared to wild-type mice. Further, Kumagai *et al.* [38] reported that estradiol restores glucose uptake, glycogen synthesis, insulin sensitivity and sex hormones, which strongly influence body fat distribution and adipocyte differentiation. These studies further suggest the role of ERα in regulating various aspects of glucose and lipid metabolism that influence the body weight.

Unambiguously, estradiol and ER were related to the changes in uterine weight [39]. We also examined the uterine weight in the present study (Figure 1B) and determined that it significantly decreased in OVX rats compared to the sham (*p* < 0.05). The results were found to be in agreement with the earlier reports by Hidaka *et al.* [40], where ovariectomized rats were shown to exhibit diminished uterine epithelial cell proliferation. Estradiol induces cell proliferation in the uterine epithelium of humans and mice by signaling through its transcription factor receptor (ERα) [41], which further concludes the positive effects of estradiol on uterine size and cell proliferation [42]. Therefore, the subsequent drop in estrogen levels following the ovariectomy accelerates the uterine weight loss. However, HRV-treated rats significantly gained uterine weight compared to OVX rats. RC is known to increase the level of estrogen and support ovarian follicle development [43]. We can thus assume that RC vinegar intake or treatment may positively affect uterine growth and development in ovariectomized rats.

We further examined the biochemical markers for liver malfunctions, as well as for bone formation and resorption in serum and urinary samples (Table 1). First, serum AST and ALT were measured to assess liver health, wherein the elevated levels indicate probable medication-induced toxicity [44]. Our data showed that the levels of AST and ALP marginally increased in OVX rats in comparison to the RC vinegar- (LRV and HRV) or alendronate (ALEN)-treated rats. However, the levels of liver-associated lipid biomarkers were not significantly different among each group, indicating that RC vinegar or alendronate treatment does not affect liver damage.

Osteoblast activity is associated with the growth and development of bone, where ALP and OC serve as the indicative biomarkers, which control osteoblast activity [45]. Elevated ALP and OC levels signify the increased rate of bone turnover and commonly occur in liver and bone disorders, including bone metastasis, bone cancers, vitamin D deficiency, Paget’s disease, osteomalacia, hyperparathyroidism and fractures [46]. In this study, ALP and OC levels in OVX increased relative to sham, ALEN and RC vinegar-treated (LRV and HRV) rat groups. While this effect seems moderate in RC vinegar- (LRV and HRV) or alendronate (ALEN)-treated groups, it considerably decreases the bone turnover. Some studies have reported a slight decrease in the positive effect of alendronate [47,48].

Furthermore, we determined the recovery effects of RC vinegar on the structure and density of trabecular and cortical bone for experimental rat groups through micro-CT images (Figure 2A,B). In the ovariectomized rat groups, BV/TV, Tb.N and BMD levels were significantly lower (*p* < 0.05) than those in the sham, whereas those of Tb.Sp, Tb.Pf and SMI (*p* < 0.05) were significantly higher. The results further suggest the comparable efficacy of RC vinegar treatment with that of the drug alendronate. Alendronate has several established activities, such as influencing the bone mass, bone turnover and bone volume and strength, which together alter the cycle of bone formation and breakdown in the body [49]. Alendronate has earlier been reported to attenuate bone loss while increasing the bone mass, which may help maintain strong bones and reduce the risk of fractures or breaks [50].

In addition, vinegar is a rich source of minerals, such as calcium, manganese and magnesium, which are important in sustaining optimal bone mass. Moreover, the acetic acid content in vinegar has also been reported to promote the absorption and retention of calcium [51]. Hence, it is generally assumed that dietary vinegar might be helpful in improving the osteoporotic condition [52]. Here, the study further finds its course to examine the functional properties of RC vinegar as a potential dietary supplement to counter the progression of osteoporosis. Therefore, we investigated the influence of RC vinegar treatments on the ovariectomized rats using GC-TOF-MS and UPLC-Q-TOF-MS, where a range of plasmatic metabolites were considered to evaluate its health benefits (Table 2 and Table 3). The metabolites differentially produced in each experimental rat groups were further examined using the PLS-DA score plots (Figure 3), followed by analyses for the statistically-significant variables (VIP > 0.7) as indicated in the metabolomic data. The present work comprehensively highlights the plasma profiles for different classes of metabolites in experimental rat groups (Table 2 and Table 3). Among the different metabolite classes shown in Table 2, phenylalanine (**6**) and tryptophan (**7**) act as important neurotransmitter precursors [53]. Phenylalanine levels and its associated genetic disorders (PKU (phenylketonuria)) have more recently been correlated with a number of osteoclasts and bone resorption activities [54]. Tryptophan is also reported to influence bone histomorphometry (wall thickness and trabecular thickness) and bone mineral density (BMD), which together suggest a bottleneck link between these essential amino acids (Phe, Trp) and osteoblast-osteoclast functions [55]. Assadi-Porter *et al.* [56] have reported that, compared to the sham, OVX animals had a low level of valine, tryptophan, serine and threonine and a high level of phenylalanine. In our study, the levels of phenylalanine and tryptophan significantly decreased in OVX rats, while they were considerably high in RC vinegar- (LRV and HRV) and alendronate (ALEN)-treated groups (Figure 4A). In recent times, some studies have further indicated these amino acids as anti-osteoporosis biomarkers, suggesting the possible pathways for conjecturing the aromatic amino acids (Phe, Trp) and the functional biochemistry of osteoblasts [57]. Additionally, phenylalanine and tryptophan are also known to interact with the calcium-sensing receptor (CaSR), and, hence, affect the body calcium metabolism and bone homeostasis [58]. Therefore, the selected class of amino acids may also serve as an essential biomarker to index and influence the bone health in experimental studies.

Additionally, among the organic acids, butyric acid (**9**) is an important member of the fatty acid subgroup called short-chain fatty acid (SCFA). SCFA has anti-inflammatory effects and the potency to prevent infiltration of immune cells in the bloodstream [59]. Fruit vinegar has also been described to contain various minerals, vitamins, complex carbohydrates and fiber, pectin, amino acids and numerous beneficial enzymes. Especially, fiber-rich foods are also abundant in SCFA [60]. The data shows that butyric acid content significantly decreased in OVX rats relative to the sham; however, RC vinegar and ALEN moderately complemented the butyric acid dearth (Figure 4B).

Further evaluation of the plasma sugars showed that the glucose (**13**) level significantly increased in all ovariectomized groups compared to that in the sham, as reported earlier by Chau *et al.* [61] in postmenopausal women. Elevation of blood glucose concentration is often related to diabetic bone loss with increased fracture risks and osteoporosis. However, the RC vinegar-treated groups (LRV and HRV) managed to maintain moderately lower glucose levels than OVX rats (Figure 4C), the reduction in glucose levels was not significantly different from that observed for OVX rats. The results positively encourage RC vinegar treatments to ovariectomized rats groups to overcome high sugar-mediated bone loss.

In this study, lysoPC 16:0 (**12**), lysoPC 18:0 (**15**), lysoPC 20:4 (**10**) and lysoPC 22:6 (**9**) were detected as the significantly-different biomarker metabolites between each rat groups (Table 3). Lysophosphatidylcholines (LPC) is a bioactive pro-inflammatory lipid generated through pathological activities, which can serve as a clinical diagnostic indicator to reveal some pathophysiological states [62]. LPC compounds are derived from the hydrolysis of phosphatidylcholines (PC) through the phospholipase A2 activity, an enzyme activated under inflammatory conditions, such as ovariectomy-induced osteoporosis [63]. In OVX rats, the level of lysoPC 22:6 (**9**) decreased, while that of lysoPC 14:0 (**5**), lysoPC 16:0 (**12**), lysoPC 16:1 (**8**), lysoPC 18:0 (**15**), lysoPC 18:3 (**7**), lysoPC 20:4 (**10**) and lysoPC 20:5 (**6**) increased compared to the sham. Many studies showed lysoPCs as significant biomarkers of anti-osteoporosis [64], bone health and bone formation [65]. Concomitantly, Liu *et al.* [57] have reported that the lysoPC level increases significantly in osteoporotic rat plasma, where the production of reactive oxygen species (ROS) overwhelms the natural antioxidant defense mechanisms. In such cases, oxidative stress may bring about extensive bone loss and skeletal fragility, resulting in acute osteoporotic conditions. Furthermore, the elevated levels of protein carbonyls in osteoporotic rats signify the disproportionate antioxidant–oxidation process resulting from the increased lysoPC contents. In comparison with the sham, the plasma levels for most of the lysoPCs increased considerably in ovariectomized rats under different experimental conditions. Unambiguously, the elevated lysoPC levels may further manifest to enhance the ROS-mediated oxidative stress, as described earlier.

## 4. Experimental Section

### 4.1. Reagents

Acetonitrile, water and methanol were purchased from Fisher Scientific (Pittsburgh, PA, USA). Methoxyamine hydrochloride, pyridine, *N*-methyl-*N*-(trimethylsilyl) trifluoroacetamide (MSTFA) and formic acid were obtained from Sigma-Aldrich (St. Louis, MO, USA). All chemicals and solvents were of analytical grade.

### 4.2. Experimental Design and Diet

The experimental protocol was approved by the Animal Care and Use Review Committee of Kyung Hee University (Protocol Number KHUASP (SE)-14-018). A total of 40, five-week-old female Sprague-Dawley rats (weighing 120–130 g) were purchased from DBL, Inc. (Eumseong, Korea). Rats were housed in polycarbonate cages and acclimatized in temperature-controlled rooms (22 ± 2 °C) with relative humidity of 55% ± 5% and 12 h of light/dark cycle alternatively, with two rats lodged in each cage. The rats were fed a standard research diet, American Institute of Nutrition (AIN)-93G (Research Diet, New Brunswick, NJ, USA), and were given water *ad libitum* for an adaptation period of 3 days.

After adaptation, five-week-old female SD rats were anesthetized with 2% of isoflurane, and ovaries were removed bilaterally. A sham operation, during which the ovaries were just touched with forceps, was performed on the sham group. One week after surgery, rats were divided into five treatment groups: (1) sham-operated control (sham); (2) ovariectomized (OVX) rats without treatment; (3) OVX rats treated with low-dose RC vinegar (LRV; 1.3% vinegar 5 mg/kg B.W./day); (4) OVX rats treated with high-dose RC vinegar (HRV; 5.2% vinegar 5 mg/kg B.W./day); and (5) OVX rats treated with alendronate (ALEN; alendronate sodium trihydrate 5 mg/kg B.W./day). All groups were treated for ten weeks. During the 10-week period, body weight was were measured weekly. The uterus were removed and adhering fats were trimmed away. At the end of the experimental period, uterus weight was measured using a balance.

### 4.3. Analysis of Serum and Urine Parameters

Blood was collected at the end of the experiment after a 12-h overnight fast. The rats were lightly anesthetized with ethyl ether, and blood samples were taken by heart puncture. Blood samples were immediately collected in serum-separating tubes (SST) and were centrifuged at 3000 rpm for 15 min at 4 °C. The top layer (serum) was stored at −70 °C until use in assays. Serum levels of osteocalcin (OC) and alkaline phosphatase (ALP) were measured using a rat osteocalcin EIA kit (Biomedical Technologies Inc., Boston, MA, USA) and an ALP estimation kit (Roche, Mannheim, Germany), respectively. For collecting the 12-h urine sample at the end of the experiment, the animals were housed in metabolic cages. During the sample collection period, the rats were restricted from their diet to avoid contamination of the urine; however, they were allowed free access to water. Instruments used for collecting urine were washed with 0.1 N HCl to prevent sample contamination. Urine samples were centrifuged at 2000 rpm for 15 min at room temperature, and the top layer was collected and stored at −70 °C until analysis. Urinary deoxypyridinoline (DPD) concentration was determined using a commercial kit, MicroVue DPD EIA (Quidel, San Diego, CA, USA). *N*-terminal telopeptides (NTx) were estimated using Urine ELISA, OSTEOMARK^®^ NTx (Wampole Laboratories, Cranbury, NJ, USA), and creatinine concentrations were determined using a commercial kit, CRE2U (Roche, Mannheim, Germany). All of the measurements of urinary bone markers were corrected for urinary creatinine and expressed as ratios.

### 4.4. Determination of Architectural and Mineralization Parameters by Using Micro-Computed Tomography

The specimens were analyzed using the SkyScan 1076 (SkyScan, Kontich, Belgium) micro-computed tomography (μCT) system. The cortical and trabecular microstructure and a 3D image of the right femur were analyzed using the SkyScan 1076 *in vivo* μCT system at 50 kV, 200 μA at a rotation step of 0.4°. Cross-sections were reconstructed using NRecon con-beam algorithm software (SkyScan), and the files were imported into CTAn software (SkyScan, Kontich, Belgium) for 3D analysis and image generation. The following parameters were measured: bone volume to tissue volume fraction (BV/TV), bone surface to volume fraction (BS/BV), trabecular thickness (Tb.Th), trabecular number (Tb.N), cortical thickness (Cs.Th), trabecular separation (Tb.Sp), trabecular pattern factor (Tb.Pf), structure model index (SMI), mean polar moment of inertia (MMI) and bone mineral density (BMD).

### 4.5. Instrumental Analysis

#### 4.5.1. Sample Preparation for MS Analysis

The plasma sample extraction was performed by adding 900 μL of ice-cold methanol to 300 μL of rat plasma at an optimal ratio of 1:3. The mixture was homogenized (30 frequency) for 3 min by using a mixer mill (Retsch GmbH & Co, Haan, Germany) and was kept at −20 °C for 1 h. Then, the sample was centrifuged at 4 °C and 12,000 rpm for 10 min. The supernatants were further passed through a 0.2-μm PTFE filter and finally transferred to Eppendorf tubes. One milliliter of the supernatant was completely dried with a speed vacuum machine (Biotron, Seoul, Korea). The dried sample was dissolved in methanol and syringe-filtered (0.2 μm) prior to the UPLC-Q-TOF-MS and GC-TOF-MS analyses. For GC-TOF-MS analysis, a two-stage chemical derivatization for the samples was performed. Firstly, oximation was carried out using 50 μL of methoxyamine hydrochloride (20 mg/mL in pyridine; for 90 min; 30 °C) followed by silylation using 50 μL of *N*-Methyl-*N*-(trimethylsilyl) trifluoroacetamide (MSTFA) (for 30 min; 37 °C).

#### 4.5.2. GC-TOF-MS Analysis

The GC-TOF-MS analyses were performed using an Agilent 4890 GC system (Palo Alto, CA, USA) coupled with a Leco TOF Pegasus III mass spectrometry. A DB-5MS capillary column (30 m length × 0.25 mm i.d. × 0.25 μm film thickness, J & W Scientific, Folsom, CA, USA) was used with a helium gas flow of 1.5 mL/min. A total of 1 μL of the derivatized sample was injected in split mode (10:1). The oven temperature was maintained at 75 °C for 2 min, then increased to 300 °C at a rate of 15 °C/min and held for 3 min. The mass data were collected in the electron ionization (EI) mode with ionization energy of 70 eV and mass scan (*m*/*z*) range of 45–1000 at an acquisition rate of 20 spectra/s. The injector and ion source temperatures were set at 250 and 230 °C, respectively.

#### 4.5.3. UPLC-Q-TOF-MS Analysis

The UPLC-Q-TOF-MS analyses for the plasma samples were performed on a Waters Micromass Q-TOF Premier with UPLC ACQUITY™ System (Waters, Milford, MA, USA) fitted with a binary solvent manager (pump) and a sample manager (auto sampler). The chromatographic operations were performed using the ACQUITY™ UPLC BEH C18 column (100 × 2.1 mm, 1.7 μm, Waters) followed by absorbance measurements using an ACQUITY™ UPLC Tunable UV (TUV) Detector (Waters). The mobile phase consisted of water (A) and acetonitrile (B) with 0.1% formic acid (*v*/*v*) at a flow rate of 0.3 mL/min. The solvent gradient condition was initially set at 5% Solvent B for 1 min followed by a linear gradient over 10 min ending at 100% Solvent B. The injection volume of samples was 5 μL, and the flow rate was maintained at 0.3 mL/min. The full-scan mass spectral range was 100–1000 *m*/*z*. The source temperature was 100 °C. The desolvation gas (nitrogen) and cone gas (nitrogen) flow rates were set at 700 L/h and 0.0 L/h, respectively, at 300 °C. The capillary and cone voltages were set to 3.0 kV and 40 V, respectively.

### 4.6. Data Processing and Statistical Analysis

The raw data from GC-TOF-MS and UPLC-Q-TOF-MS acquisitions were converted to NetCDF format (*.cdf) using ChromaTOF (LECO) and DataBridge (Waters) software, respectively. Subsequently, the netCDF files were aligned with the MS, data using the Metalign software package (http://www.metalign.nl) [66]. The aligned *.cdv files were then transferred to Excel (Microsoft, Redmond, WA, USA) data sheets for sequential multivariate and statistical analyses using theSIMCA-P+ 12.0 software program (Umetrics, Umea, Sweden). Principal component analysis (PCA) was performed to obtain an overview of the MS data for metabolites and to identify the singularity value. Further, the partial least squares-discriminant analysis (PLS-DA) was applied to find a model that separated classes of observations of the *Y* axis (or vertical line) based on their *X* variables (or horizontal line). The PLS-DA model performance was based on the cumulative goodness of fit (R^2^) and the cumulative goodness of prediction (Q^2^) for each model. Metabolites with a variable importance projection (VIP) value greater than 0.7 and a *p*-value less than 0.05 were selected as potential metabolites, each related to a different group. The *p*-values for different metabolite-based cluster groups were determined using Statistica 7 (StatSoft, Tulsa, OK, USA). Experimental groups were compared using one-way ANOVA and Duncan’s test, with *p*-values <0.05 considered significant. All histological data were analyzed using PASW Statistics 18 software (SPSS, Chicago, IL, USA), and *p* < 0.05 (two-tailed) was considered statistically significant. After the multivariate statistical analysis, major metabolites were positively identified by comparing the mass spectra and retention times with those of standard compounds based on databases from the NIST mass spectral database (National Institute of Standards and Technology, FairCom, Gaithersburg, MD, USA), in-house library and references. In UPLC-Q-TOF-MS analysis, the significantly-different metabolites were matched with the references and the Human Metabolome Database (HMDB) [67].

## 5. Conclusions

Our study evaluated the effect of RC vinegar on ovariectomy-induced osteoporosis in rats by employing biochemical and metabolomic methods, including GC-TOF-MS and UPLC-Q-TOF-MS. Our results demonstrated that RC vinegar has a positive effect on bone mass, bone resorption and bone strength in experimental rat groups under controlled conditions. We further established that oral administration of RC vinegar decreased the BMD loss, which was accompanied by the reduction in serum ALP and OC levels and a consequent bone volume increase in treated rats (HRV and LRV) compared to OVX.

The study provides valuable insight into the RC vinegar-mediated reduction in bone loss and bone turnover rates in ovariectomized rats, mimicking the human postmenopausal conditions.

Additionally, eight potential metabolites, including butyric acid, phenylalanine, glucose, tryptophan and lysoPC 16:0, lysoPC 18:0, lysoPC 20:4, and lysoPC 22:6, were identified as osteoporosis hallmarks in ovariectomized rat groups. Moreover, the study further highlights the precedence of MS-based analytical and multivariate (PLS-DA) data interpretation methods towards the precise and rapid interpretation of colossal metabolomics data. The present methodology can further be anticipated to address the dietary or health food correlations with an organisms’ “-omics” and health phenotypes.

## Supplementary Materials

Supplementary materials can be accessed at: http://www.mdpi.com/1420-3049/21/2/149/s1.
