# Supplementary material for: Metabolite Profiling Reveals the Effect of Dietary Rubus coreanus Vinegar on Ovariectomy-Induced Osteoporosis in a Rat Model
Source: Molecules. 2016 Jan 26;21(2):149. doi: 10.3390/molecules21020149 (PMC6273122; doi:10.3390/molecules21020149)
Supplement: Supplementary file 1 [file molecules-21-00149-s001.pdf]

Supplementary Materials: Metabolite Profiling Reveals the Effect of Dietary *Rubus coreanus* Vinegar on Ovariectomy-Induced Osteoporosis in a Rat Model

Mee Youn Lee, Hyang Yeon Kim, Digar Singh, Soo Hwan Yeo, Seong Yeol Baek, Yoo Kyoung Park and Choong Hwan Lee

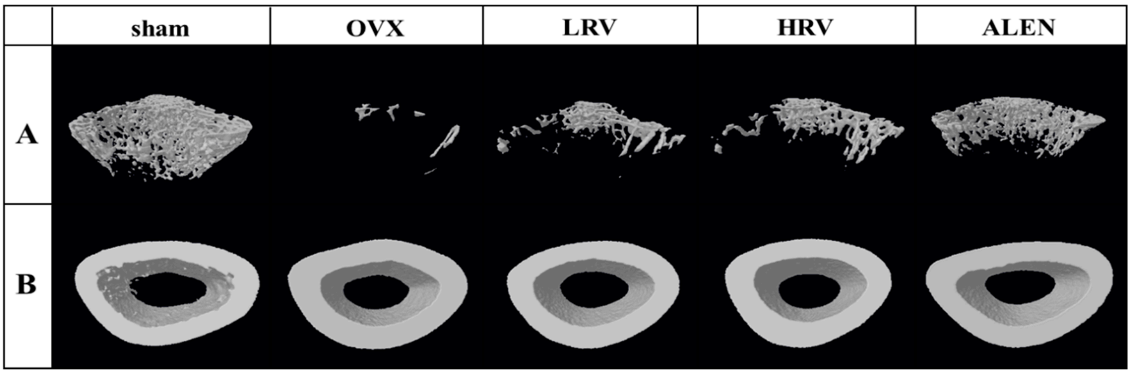

Figure S1. Representative μCT images of the trabecular bone (**A**) and the cortical bone (**B**). (From left to right) sham, sham-operated control; OVX, ovariectomized rat without treatment; LRV, ovariectomized at treated with low-dose RC vinegar; HRV, ovariectomized rat treated with high-dose RC vinegar; ALEN, ovariectomized rat treated with alendronate.

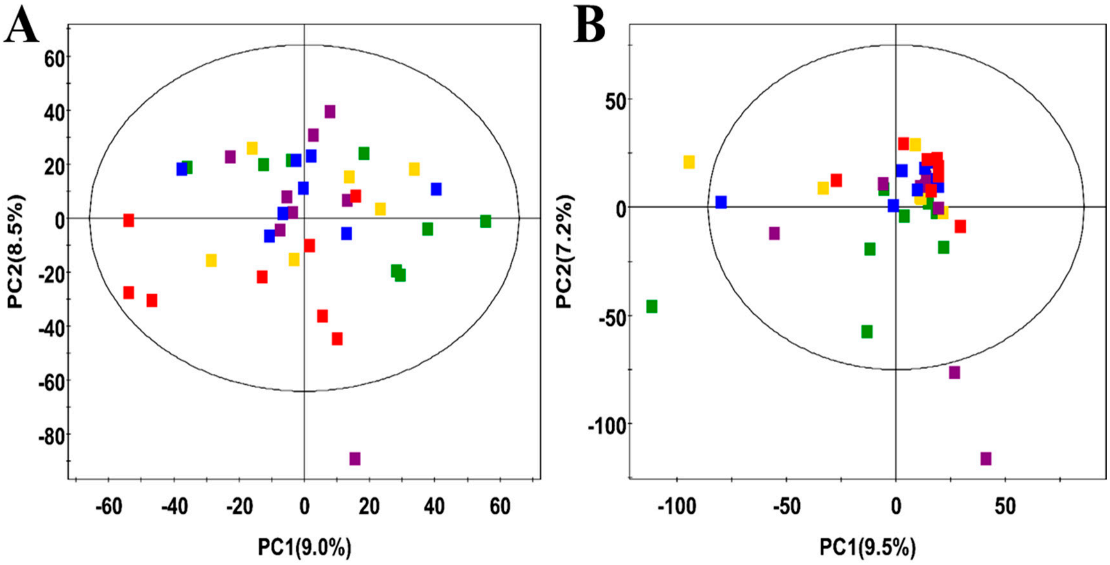

Figure S2. Principal component analysis (PCA) score plots derived from GC-TOF-MS (**A**) and UPLC-Q-TOF-MS (**B**) in ovariectomized rat groups. (■; sham-operated control, ■; ovariectomized rat without treatment, ■; ovariectomized rat treated with low-dose RC vinegar, ■; ovariectomized rat treated with high-dose RC vinegar, ■; ovariectomized rat treated with alendronate).
